# Prevalence and risk factors of anaemia in hospitalised HIV-infected patients in southeast China: a retrospective study

**DOI:** 10.1017/S0950268818003618

**Published:** 2019-01-30

**Authors:** J. L. Lai, Y. H. Chen, Y. M. Liu, J. J. Yuan, J. Lin, A. Q. Huang, H. H. Ye

**Affiliations:** Department of Infectious Diseases, Mengchao Hepatobiliary Hospital of Fujian Medical University, Infectious Disease Hospital of Fuzhou, Fuzhou, China

**Keywords:** Anaemia, HIV, opportunistic infection, prevalence, risk factors

## Abstract

The association between opportunistic infection (OI) and anaemia among HIV-infected patients remains to be studied. We investigated the prevalence and risk factors of anaemia in hospitalised HIV-infected patients to reveal the association between OI and anaemia. We conducted a retrospective study of HIV-positive hospitalised patients from June 2016 to December 2017 in Mengchao Hepatobiliary Hospital of Fujian Medical University. Patients’ information on socio-demographic and clinical characteristics were carefully collected. The comparison of anaemia prevalence between groups was conducted with *χ*^2^ test. A logistic regression model was carried out to analyse the predictors of anaemia. The total prevalence of anaemia in hospitalised HIV-infected patients was 55.15%. The prevalence of mild, moderate and severe anaemia was 41.42%, 11.08% and 2.64%, respectively. Predictors independently associated with anaemia were: CD4 counts <50 cells/μl (odds ratio (OR): 6.376, 95% confidence interval (CI) = 1.916–21.215, *P* = 0.003), CD4 counts 50–199 cells/μl (OR: 6.303, 95% CI = 1.874–21.203, *P* = 0.003), co-infection with tuberculosis (TB) (OR: 2.703, 95% CI = 1.349–5.414, *P* = 0.005) or Penicillium marneffei (PM) (OR: 7.162, 95% CI = 3.147–15.299, *P* < 0.001). In Fujian, China, more than half inpatients with HIV were anaemic, but severe anaemia is infrequent. Lower CD4 counts, co-infection with TB or PM were independent risk factors for anaemia. Chinese HIV patients especially with TB, PM infection and low CD4 level should be routinely detected for anaemia to improve therapy.

## Background

Anaemia is a major complication in HIV patients and leads to a poor quality of life, progression of the HIV disease, shorter life expectancy [1]. Survival time and prognosis in HIV-infected patients could be improved with anaemia correction [2–4].

Due to different study settings, the prevalence of anaemia in the HIV patients ranges from 20% to 84% [5–7] in the world and the risk factors are also different including sex, age, educational status, antiretroviral therapy (ART) status, stage of HIV disease, CD4^+^ T lymphocyte count, HIV RNA loads and presence of opportunistic infection (OI) [7–10]. In China, the prevalence of anaemia ranges from 9.8% to 51% in various regions depending on different geographical-social-economic conditions. Dai *et al*. [11] analysed data from 3452 ART-naive HIV-infected patients of Beijing Ditan Hospital in China, and revealed that the overall prevalence of anaemia was only 9.8%. They found that female, older age, lower body mass index (BMI) and higher load of HIV RNA were associated with a higher prevalence of anaemia. While Shen and colleagues [12] reported that the overall prevalence of anaemia among Chinese adults newly diagnosed with HIV-infection was 51.9%. In these cases, anaemia was associated with minority ethnicity, older age and lower CD4 counts. Another study [13] indicated that 38.9% of HIV-infected individuals in Xinjiang, China, were anaemic at the initiation of ART. It suggested that Uyghur ethnicity, female, lower CD4 counts, lower BMI, self-reported tuberculosis (TB) infection and oral candidiasis were the risk factors of anaemia.

Despite the considerable literature on prevalence and risk factors of anaemia in HIV patients, there have been few studies about the association between OI (especially TB, Penicillium marneffei (PM)) and anaemia among inpatients with HIV. OI such as TB, PM and cytomegalovirus (CMV) is becoming an important cause of hospitalisation and mortality in the era of ART. OI can affect the treatment route of patients. Therefore, it is critical to identify co-infection in patients with HIV. Therefore, this study was aimed at analysing the prevalence of anaemia and its risk factors especially OI among inpatients with HIV in Mengchao Hepatobiliary Hospital of Fujian Medical University, the largest specialised hospital for HIV-infected patients in Southeast China.

## Methods

### Ethical statement

This study was approved by the Ethics Committee of Mengchao Hepatobiliary Hospital of Fujian Medical University. Existing routine clinical and therapeutic data were anonymously used and were abstracted from the electronic medical records. So, the need to obtain informed consent was waived.

### Study design and patient selection

We performed a retrospective study of hospitalised patients with HIV in Mengchao Hepatobiliary Hospital of Fujian Medical University, the largest designated HIV/AIDS care hospital in southeast China between June 2016 and December 2017. Individuals who were adult (18 years or above), ART-naïve and HIV-positive were included. Patients with cirrhosis, incomplete data, HIV RNA <250 IU/ml and pregnant women were excluded. All patients were confirmed by enzyme-linked immunosorbent assay and Western blot testing laboratory detection to be positive for HIV antibody and the diagnosis was in line with national HIV diagnostic criteria.

### Data collection and definitions

Patients’ data on socio-demographic variables, clinical characteristics and laboratory data were checked item by item after abstraction. All the study laboratories successfully completed a standardisation and certification programme. CD4 counts were measured using the BD facscount system (Becton Dickenson, California, USA). Total white blood counts (WBC), haemoglobin (HB) and platelets (PLT) were analysed using a haematology analyser (Sysmex, Kobe, Japan). Plasma HIV RNA levels were analysed using the Ampliform HIV-1 Monitor Test, version 1.5 (Roche, Basel, Switzerland), with a detection limit threshold was <250 IU/ml.

Anaemia was diagnosed as a HB level <120 g/l (men) and <110 g/l (women). Anaemia status was categorised as follows: mild anaemia (HB 90–119 g/l (men) or 90–109 g/l (women)), moderate anaemia (HB 60–89 g/l) and severe anaemia (HB <60 g/l). WBC <4.0 × 10^9^/l and PLT <100 × 10^9^/l were diagnosed as leucopaenia and thrombocytopaenia respectively in peripheral blood.

The definition of TB was based on the World Health Organization (WHO) guidelines [14]. The diagnosis of TB was confirmed when mycobacterium TB was positive in microbiological sample. TB was considered as the probable diagnosis when a patient showed symptoms of TB (fever, night sweats, cough, haemoptysis, chest pain and weight loss), a positive interferon-γ release assay or tuberculin skin test and an abnormal chest radiograph or extrapulmonary imaging associated with improvement after anti-TB treatment. Patients with latent tuberculosis infection were excluded from this study.

PM was diagnosed based on isolation and identification of PM from any microbiological sample. Cryptococcosis was initially screened by detection of serum cryptococcal antigen. Patients with symptoms (signs) of cryptococcal meningitis and positive serum cryptococcal antigen should undergo a lumbar puncture with CSF examination, India ink or CSF cryptococcal antigen assay. CMV viraemia was based on positive CMV-DNA or pp65 antigen.

### Statistical analysis

All data were analysed using SPSS 16.0 (SPSS Inc., Chicago, USA). Continuous variables were expressed as means ± S.D. if normally distributed, otherwise presented as median (interquartile range). Categorical variables were indicated as numbers (percentage). The comparison of anaemia prevalence between groups was conducted with Pearson *χ*^2^ test, or adjust *χ*^2^ test or Fisher’ exact test, as appropriate. To exclude the effects of potential confounders, we used a logistic regression and backward stepwise methods to analyse the association between risk factors and anaemia. The odds ratio (OR) and 95% confidence intervals (CIs) were calculated. Variables included in the model were sex, age, CD4 count, HIV RNA loads, CMV, Epstein–Barr virus (EBV), TB, PM and cryptococcosis which were based on biological plausibility and epidemiological importance. A threshold of *P* < 0.05 indicates statistical significance (two-sided).

## Results

### Patient characteristics

There were total 462 HIV-positive patients recruited of which 29 with incomplete data, 26 ARV-experienced, 18 with HIV RNA <250 IU/ml, six with cirrhosis, four were aged below 18. Finally, 379 patients were enrolled. The age of the study population was between 18 and 87 years with mean age (42 ± 0.8) years (41 years for male, 48 years for female). There were 317 males (83.6%) and 62 females (16.4%). About 83.1% patients had a baseline CD4 counts <200 cells/μl and 78.1% with HIV RNA loads >1 × 10^4^ IU/ml. The basic characteristics of the study subjects are described in Table 1.

**Table 1.** Clinical characteristics of patients with HIV infection

Characteristics	Patients no. (%)
Sex	
Male	317 (83.6)
Female	62 (16.4)
Age, years	
18–39	190 (50.1)
40–59	128 (33.8)
⩾60	61 (16.1)
CD4 counts, cells/μl	
Median CD4 counts (IQ)	38 (128)
<50	211 (55.7)
50–199	104 (27.4)
200–350	40 (10.6)
>350	24 (6.3)
HIV RNA, IU/ml	
<1 × 10^4^	83 (21.9)
1 × 10^4^–1 × 10^6^	213 (56.2)
⩾1 × 10^6^	83 (21.9)
WBC counts	
<4 × 10^9^	135 (35.6)
⩾4 × 10^9^	244 (64.4)
PLT counts	
<100 × 10^9^	64 (16.9)
⩾100 × 10^9^	315 (83.1)
CMV	
Positive	148 (39.1)
Negative	231 (60.9)
EBV	
Positive	39 (10.3)
Negative	340 (89.7)
Tuberculosis	
Positive	52 (13.7)
Negative	327 (86.3)
Penicillium marneffei	
Positive	61 (16.1)
Negative	318 (83.9)
Cryptococcosis	
Positive	50 (13.2%)
Negative	329 (86.8)

IQ, interquartile range; WBC, white blood count; PLT, platelets; CMV, cytomegalovirus; EBV, Epstein–Barr virus.

### Prevalence of anaemia in patients with HIV

Among 379 patients, 209 (55.15%) had anaemia. The overall prevalence of mild, moderate and severe anaemia was 41.42%, 11.08% and 2.64%, respectively. About 55.52% of male patients and 53.23% of female patients were anaemic (Table 2).

**Table 2.** Prevalence of anaemia in 379 patients with HIV infection stratified by variables

		Anaemia (*n*, %)			Without			
Variable		Mild	Moderate	Severe	Anaemia (*n*, %)	^a^*P*	OR	95% CI
Sex	Male	139 (43.85)	28 (8.83)	9 (2.84)	141 (44.48)	0.74	1.097	0.636–1.893
	Female	18 (29.03)	14 (22.58)	1 (1.61)	29 (46.77)		1	
CD4 counts (cells/μl)	<50	89 (42.18)	36 (17.06)	4 (1.90)	82 (38.86)	<0.001	7.866	2.596–23.835
	50–199	46 (44.23)	6 (5.80)	6 (5.80)	46 (44.23)	0.001	6.304	2.014–19.735
	200–350	14 (35.00)	3 (7.50)	0 (0)	23 (57.50)	0.033	3.696	1.066–12.811
	>350	3 (12.50)	1 (4.17)	0 (0)	20 (83.33)		1	
TB	Positive	28 (53.85)	6 (11.54)	2 (3.85)	16 (30.77)	0.025	2.028	1.082–3.798
	Negative	123 (37.61)	40 (12.23)	9 (2.75)	155 (47.40)		1	
PM	Positive	33 (54.10)	18 (29.51)	2 (3.28)	8 (13.11)	<0.001	7.055	3.25–15.318
	Negative	118 (37.12)	28 (8.81)	8 (2.52)	164 (51.57)		1	
EBV	Positive	25 (64.10)	5 (12.82)	1 (2.56)	8 (20.51)	0.001	3.569	1.594–7.988
	Negative	127 (37.35)	41 (12.06)	9 (2.65)	163 (47.94)		1	
HIV RNA (IU/ml)	>1 × 10^6^	35 (42.19)	10 (12.05)	4 (4.82)	34 (40.96)	0.03	1.976	1.066–3.665
	1 × 10^4^–1 × 10^6^	96 (45.07)	27 (12.68)	2 (0.94)	88 (41.31)	0.01	1.948	1.165–3.257
	<1 × 10^4^	22 (26.51)	9 (10.84)	4 (4.82)	48 (57.83)		1	
CMV	Positive	66 (44.59)	18 (12.16)	4 (2.70)	60 (40.54)	0.152	1.357	0.894–2.060
	Negative	86 (37.23)	28 (12.12)	6 (2.60)	111 (48.05)		1	
Cryp	Positive	17 (34.00)	5 (10.00)	1 (2.00)	27 (54.00)	0.176	0.663	0.365–1.205
	Negative	135 (41.03)	41 (12.46)	9 (2.74)	144 (43.77)		1	
Age (years)	>60	33 (54.10)	5 (8.20)	1 (1.64)	22 (36.07)	0.049	1.810	0.999–3.282
	40–59	51 (39.84)	19 (14.84)	5 (3.91)	53 (41.41)	0.110	1.445	0.919–2.272
	18–39	68 (35.79)	22 (11.58)	4 (2.11)	96 (50.53)		1	

TB, tuberculosis; PM, Penicillium marneffei; EBV, Epstein–Barr virus; CMV, cytomegalovirus; Cryp, Cryptococcosis; ^a^*P*, anaemia cases were compared to without anaemia cases; OR, odds ratio; anaemia proportion to proportion without anaemia; CI, confidence interval.

The prevalence of anaemia was 16.67%, 42.50%, 55.77% and 61.14% in patients with a CD4 counts of >350, 200–350, 50–199 and <50 cells/μl, respectively, increasing with decreasing CD4 counts (*P* = 0.033, *P* = 0.001, *P* < 0.001). The prevalence of mild anaemia and of moderate anaemia showed an increasing trend with decreasing CD4 counts (Table 2).

The prevalence of anaemia in patients with TB was 69.23%, which was higher than patients without TB (52.60%, *P* = 0.025). Individuals with TB were more likely to occur mild anaemia than patients without TB. Patients with PM had significantly higher prevalence of anaemia than patients without PM (*P* < 0.001). Mild anaemia and moderate anaemia were found to be more common in patients with PM compared to patients without PM. There was also significant difference on the prevalence of anaemia between patients with EBV and without EBV infection (*P* = 0.001). Mild anaemia occurred in more patients with EBV (64.10%) than patients without EBV (37.35%) (Table 2).

The prevalence of anaemia gradually increased from 42.17% to 59.04% in patients with increasing HIV RNA loads (*P* = 0.01, *P* = 0.03). The prevalence of mild anaemia was lower in patients with HIV RNA <1 × 10^4^ IU/ml than patients with HIV RNA 1 × 10^4^–1 × 10^6^ IU/ml and >1 × 10^6^ IU/ml. While, the prevalence of moderate anaemia in patients with HIV RNA 1 × 10^4^–1 × 10^6^ IU/ml was lower than those patients with HIV RNA <1 × 10^4^ IU/ml and >1 × 10^6^ IU/ml. The prevalence of anaemia increased (49.47%, 58.59%, 63.93%) with increasing age (18–39, 40–59 and ⩾60 years, respectively) (*P* = 0.110, *P* = 0.049) (Table 2).

### Risk factors for anaemia among hospitalised HIV-infected patients

We analysed risk factors associated with the incidence of anaemia by using a logistic regression model. Table 3 describes the results of the final regression model. CD4 counts <50 cells/μl (*P* = 0.003), CD4 counts 50–199 cells/μl (*P* = 0.003), co-infection with TB (*P* = 0.005) or PM (*P* < 0.001) were significantly related to an increased risk of anaemia. HIV RNA loads, age, sex and co-infection with CMV, EBV, or cryptococcus failed to show a relationship with the incidence of anaemia.

**Table 3.** Predictors of anaemia in logistic regression model

Risk factors		*P*	OR	95% CI
CD4 counts (cells/μl)	<50	0.003	6.376	1.916–21.215
	50–199	0.003	6.303	1.874–21.203
	200–350	0.072	3.287	0.9–12.004
	>350		1	
Penicillium marneffei	Positive	<0.001	7.162	3.147–16.299
	Negative		1	
Tuberculosis	Positive	0.005	2.703	1.349–5.414
	Negative		1	
Gender	Female	0.868	1.056	0.577–2.003
	Male		1	
Age (years)	18 ⩽ age < 40	0.318	0.426	0.08–2.272
	40 ⩽ age < 60	0.585	0.755	0.275–2.070
	Age ⩾ 60		1	

OR, odds ratio; CI, confidence interval.

## Discussion

In the current study, we observed the prevalence and risk factors of anaemia in hospitalised HIV-infected patients in southeast China. Our results demonstrated that the incidence of anaemia was 55.15%, with the majority of patients with mild to moderate anaemia. The independent risk factors of anaemia were lower CD4 counts, co-infected with PM or TB.

The prevalence of anaemia in our study is consistent with the results of prior research from Northeastern Nigeria (57.5%) [15], Ethiopia (52.6%) [16] and China (51.9%) [12]. Similar findings were reported from studies in Hispanics (41.5%) [10], Indonesia (49.6%) [17] and Uganda (47.8%) [18]. The prevalence of anaemia in the current study was much higher compared to two studies from Ethiopia done by Gedefaw *et al*. (23.1%) [19] and Melese *et al*. (23%) [20], respectively. However, both these studies included ART-experienced HIV patients and found that the prevalence of anaemia in ART-experienced individuals was lower than naïve individuals. Another study conducted in China reported that the prevalence of anaemia was only 9.8% among ART-naïve adult HIV patients [11]. The probable reason was that a high proportion of urban patients were included and managed in a hospital-based setting in favour of early detection of anaemia [11]. Studies from Congo (69%) [8], Ghanaian (63%) [21] and Iran (71%) [22] reported a much higher prevalence of anaemia compared to our study. However, with the exception of the socio-demographic differences, those studies either included a higher percentage of female patients or used different definition of anaemia. The current study confirmed prior research that mild to moderate anaemia was very common in patients with HIV, but severe anaemia is relatively infrequent.

Numerous studies have suggested multiple factors were associated with anaemia among HIV patients [3, 5, 20, 23–25]. Lower CD4 counts were found to be an independent risk factor of anaemia in our study. This finding was in accordance with the reports from the USA [26], Uganda [24], India [27], Mexican [28], Hispanics [10], China [11–13] and Ethiopia [7, 9, 20]. It is widely accepted that patients with lower CD4 counts have a higher risk of multiple OI such as TB, Histoplasma, PM and Leishmania which may infiltrate the bone marrow and inhibit erythropoiesis. In addition, lower CD4 means the progression of HIV disease with higher viral burden, which increased cytokine-mediated myelosuppression leading to anaemia.

Our study demonstrated that anaemia was more frequent among patients with TB than those without TB. The total prevalence of anaemia among patients with TB was 69.23% and TB was an independent risk factor for anaemia. A similar finding was demonstrated from Mert *et al*. [29] in Turkey in which 86% TB patients were anaemic. McDermid and colleagues [30] have reported that HB was significantly associated with TB even adjusting for TB susceptibility factors. A study from Southern India [31] found that TB had strong independent associations with anaemia in a multivariate model (OR: 1.6, 95% CI: 1.4–1.8). Mijiti *et al*. [13] in China described that patients with TB co-infection was associated with a higher prevalence of anaemia in both univariate analysis and multivariate logistic regression analysis. Since it is well-known TB causes anaemia through a variety of mechanisms including malnutrition, haemoptysis, anaemia of chronic illness and bone marrow infiltration. However, TB causes anaemia but why some patients do not become anaemic is unclear.

In agreement with other studies, we found the prevalence of anaemia among HIV patients with PM co-infection was up to 86.89%. Ye *et al*. [32] reported that 92.85% patients with PM were anaemic. In a retrospective analysis of 26 patients with PM infection from September 2005 to April 2014 at Fujian Provincial Hospital, China, Li *et al*. reported that 74% HIV patients with PM were anaemic [33]. However, PM is only endemic to Southeast Asia and southern part of China. These studies were case reports or descriptive researches which didn't exclude potential confounders of anaemia. In our study we found that PM was a strong independent risk factor of anaemia in patients with HIV.

Furthermore, co-infected with CMV or cryptococcosis was negatively associated with the prevalence of anaemia. This probably can be explained that most patients in our study only have CMV viraemia or serum cryptococcal antigenaemia, seldom with related end-organ diseases such as CMV retinitis (cephalitis, colitis), cryptococcal meningitis or disseminated cryptococcosis. Subbaraman *et al*. [31] also found that cryptococcal meningitis was not associated with anaemia in patients in India. Liechty *et al*. [34] reported that the prevalence of anaemia was not different between patients with serum cryptococcal antigen negative or positive.

Although the prevalences of anaemia and of mild anaemia were higher in both patients with higher HIV RNA loads and EBV co-infection, HIV RNA and EBV co-infection were not robust contributors to anaemia after controlling confounding factors in logistic regression analyses. This is in line with previous finding from Mata-Marín *et al*. [28] who reported that many factors including HIV RNA viral loads >100 000 copies/ml were associated with anaemia in a univariate analysis, but only CD4 count <200 cells/μl was associated with an increased risk of anaemia in the multivariate analysis. Studies from Santiago-Rodríguez *et al*. [10] and Dai *et al*. [11] found that higher HIV RNA load was independently relative to increased odds of anaemia, maybe due to a higher proportion of patients with higher HIV RNA loads and different HIV RNA stratified method. As to EBV, it is perhaps due to the low prevalence of EBV infection in our study (10.3%).

There is no association between anaemia and sex or age in current study. It was consistent with results from Ethiopia [9, 19, 20] and Hispanics [10], but contrary to the results from Dai *et al*. in China [11] and Subbaraman *et al*. in India [31]. Shen *et al*. [12] in China found that older age was significantly associated with an increased risk of anaemia but not sex. Contrary results may be due to different distribution of patients’ age and sex.

Finally, this study specifically assesses association between anaemia and OI in HIV-infected inpatients in China. And we found PM was an independent risk factor for anaemia in HIV-infected patients which has never been described in previous papers. The findings confirmed and highlight OI especially PM as an independent risk factors to anaemia in HIV patients.

Nonetheless, limitations should be mentioned. Since the causes of anaemia in HIV patients are multifactorial, some causes may not been identified in our study. In addition, the retrospective study limits the interpretation of the causal relationship between risk factors and anaemia. Further prospective studies should be conducted to explore the reasons of anaemia in HIV-infected patients.

## Conclusions

Our study indicated that anaemia is very common in hospitalised HIV-infected patients in southeast China. Most patients were mild to moderate anaemic. Lower CD4 count, co-infection with PM or TB were independent risk factors of anaemia in HIV-infected inpatients. Chinese HIV patients especially with PM or TB infection and low CD4 level should be routinely detected for anaemia to improve treatment.
